# Delivery of intravesical botulinum toxin A using low-energy shockwaves in the treatment of overactive bladder: A preliminary clinical study

**DOI:** 10.1080/2090598X.2019.1605676

**Published:** 2019-05-15

**Authors:** Mohammed Nageib, Ahmed S. El-Hefnawy, Mohamed H. Zahran, Nasr A. El-Tabey, Khaled Z. Sheir, Ahmed A. Shokeir

**Affiliations:** Urology and Nephrology Center, Mansoura University, Mansoura, Egypt

**Keywords:** Botulinum toxin A, overactive bladder, shockwaves

## Abstract

**Objective**: To evaluate the efficacy and safety of botulinum toxin A (BoNT-A) instillation in the bladder under the effect of low-energy shockwaves (LESWs) for the treatment of refractory idiopathic overactive bladder (OAB).

**Patients and methods**: A preliminary clinical study was conducted, including 15 patients with refractory OAB, between September 2016 and July 2017. Intravesical instillation of 100 IU of BoNT-A was done followed by LESWs (3000 shocks over 10 min) exposure to the supra-pubic area. Patients were followed-up by urine analysis, urine culture, post-void residual urine volume (PVR), and Overactive Bladder Symptom Score (OABSS) at 1, 2 and 3 months.

**Results**: There were statistically significant improvements in all OABSS domains and the total score after 1 and 2 months of treatment (*P* < 0.05). Whereas, only the nocturia domain remained significantly improved after 3 months (*P* = 0.02). There was no significant increase in PVR throughout the study period (*P* > 0.05) and none of the patients required clean intermittent catheterisation. Two, two and three patients developed urinary tract infections after 1, 2 and 3 months, respectively.

**Conclusion**: Intravesical instillation of BoNT-A and LESWs is safe and effective method for the treatment of refractory OAB with a durable response for 2 months.

**Abbreviations:** BoNT-A: botulinum toxin A; CIC: clean intermittent catheterisation; DO: detrusor overactivity; LESWs: low-energy shockwaves; OAB: overactive bladder; OABSS: Overactive Bladder Symptom Score; Q_max_: maximum urinary flow rate; QoL: quality of life; UUI: urgency urinary incontinence

## Introduction

Overactive bladder (OAB) is a condition characterised by the presence of urinary urgency, typically combined with frequency and nocturia, with or without urgency urinary incontinence (UUI), in the absence of UTI or other clear pathology [1]. Bladder and behavioural training, pharmacological treatment and surgical therapies are different treatment options for OAB [2]. The aim of the treatment is to reduce the occurrence of bothersome symptoms. Antimuscarinics are well established as pharmacotherapy for reducing OAB symptoms and improving quality of life (QoL) [2]. However, their use is limited in some patients by insufficient response to treatment, i.e. ‘refractory OAB’, or by intolerable side-effects such as dry mouth, blurred vision, constipation, and cognitive impairment [2].

After a trial of pharmacotherapy, if the patient has not had an adequate improvement in symptoms, intravesical injection of botulinum toxin A (BoNT-A) can be offered as the next step. BoNT-A is a neurotoxin, it contains a heavy chain that binds to the presynaptic terminal of the neuromuscular junction, and inhibits the release of acetylcholine from the presynaptic vesicles at the axon terminal of the motor end-plate resulting in flaccid paralysis of the muscles.

Intradetrusor injection of BoNT-A significantly improves frequency symptoms over 24 h, UUI, urodynamic changes of OAB, and QoL; together with a reduction in urgency and UI, by 80% and 60%, respectively. BoNT-A injection has a peak effect at 4 weeks lasting up to 9 months, with the possibility of repeated treatment efficacy up to 10 treatment cycles. There is a possibility of increased post-void residual urine volume (PVR), occurring in 20–40% of individuals. Some of them may need clean intermittent catheterisation (CIC) [3]. However, drug leakage outside the bladder, haematuria, pain at injection sites, and uneven distribution are also possible complications. Moreover, the procedure needs anaesthesia. So, there is an urgent need to develop a simpler and less risky method to deliver BoNT-A without the need for injection under anaesthesia [4]. Therefore, intravesical instillation rather than injection of BoNT-A seems to be a sound idea. Nevertheless, BoNT-A delivery to the bladder tissue after intravesical instillation is hampered by the urothelium’s impermeability, which results from the watertight barrier located at the umbrella cells in the superficial layers of bladder urothelium that are augmented by glycosaminoglycans and uroplakins [5].

Low-energy shockwaves (LESWs) have been reported to improve tissue regeneration and have been used for the treatment of musculoskeletal disorders, ischaemic cardiovascular disorders, and erectile dysfunction [6,7]. LESWs increase tissue permeability and drug delivery into cells by the shear force generated by the movement of liquid relative to cells, which temporarily affects the permeability of the plasma membrane. So, it can deliver macromolecular drugs into the cell cytoplasm without toxicity [8]. In a recent experimental study, LESWs temporarily increased urothelial permeability and facilitated delivery of installed intravesical BoNT-A without the need for injection [4].

In the present study, we aimed to test the safety and efficacy of using LESWs to deliver BoNT-A into the bladder tissue after being installed in the bladder in small number of patients with refractory OAB in a preliminary clinical study.

## Patients and methods

### Patients

After approval by the Local Ethics Committee (MD/16.12.24), a pilot study was conducted between September 2016 and July 2017 to assess the safety and efficacy of the delivery of intravesical BoNT-A using LESWs in the treatment of refractory OAB. The study included adult patients with idiopathic detrusor overactivity (DO) refractory to antimuscarinic treatment for at least 2 months. Patients aged <18 years, those with sensitivity to BoNT-A, and those with neurogenic DO or active UTI, were excluded from the study. Eligible patients signed a fully informed consent.

### Intervention

All patients were assessed by full history, clinical (including local and neurological) examination, Overactive Bladder Symptom Score (OABSS), urine analysis and mid-stream culture, flowmetry, post-void residual urine volume (PVR), and urodynamic study according to ICS recommendations. The OABSS includes four domains (frequency, urgency, urge incontinence, and nocturia); each given a score according to the severity of the symptoms [9].

**Figure 1.**
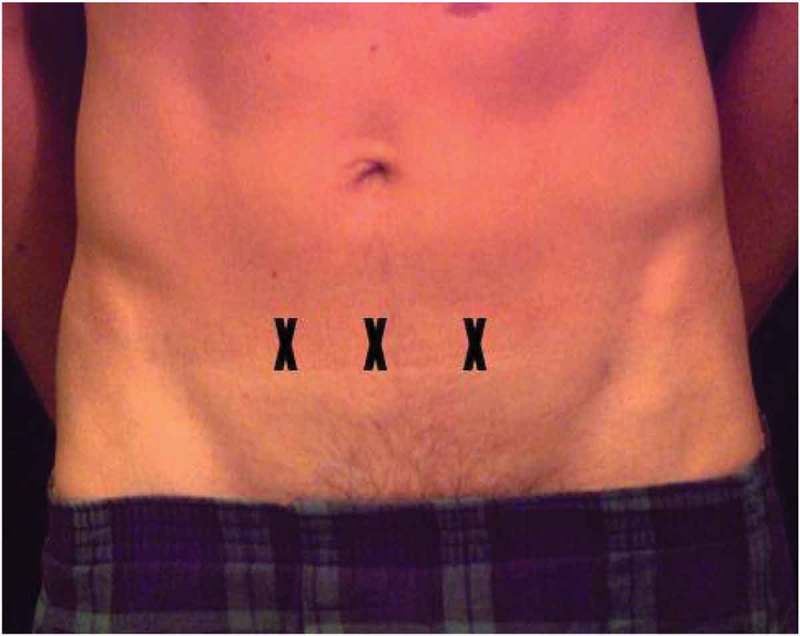
Sites of shockwaves exposure.

The procedures were done without anaesthesia or preoperative antibiotics. BoNT-A vials (Egypt – Allergan, 100 IU) were dissolved in a volume of saline equal to half of the estimated bladder capacity (from the urodynamic study) for every patient. All patients were catheterised using a 12-F Nelaton catheter until complete evacuation of the bladder, then the saline containing BoNT-A was instilled in to the bladder and the catheter was removed. Then, the patient was subjected to LESWs delivered to the suprapubic region at three horizontal points on the suprapubic transverse crease ~2 cm (1 fingerbreadth) above the pubic bone and ~3.8 cm (2 fingerbreadths) from each other in an equally distributed manner as shown in Figure 1. The target dose of LESWs was 3000 shocks with power of 6.6 mJ/shock and frequency of 300 shocks/min.

All the patients were recommended to avoid micturition for 2 h, giving a chance for BONT-A absorption, and were kept under observation for monitoring and treatment of any adverse events. Patients were advised to return in case of urine retention, haematuria, and/or fever. The follow-up schedule was 1, 2 and 3 months, with patients assessed at every visit by urine analysis, urine culture, flowmetry, PVR, and OABSS.

### Study endpoint

The primary endpoint was to evaluate the safety of the new technique. The secondary endpoint was to evaluate the impact of the new technique on the OABSS. Moreover, we evaluated the impact of the technique on the maximum urinary flow rate (Q_max_) and PVR.

### Statistical analysis

This was a self-controlled study. Data of all patients were compared before and after treatment at all time-points of follow-up. Descriptive data were expressed according to distribution. Comparison of non-parametric distributed continuous variable was done using the Mann–Whitney *U*-test. All statistical tests were done using the Statistical Package for the Social Sciences (SPSS®) version 21 (SPSS Inc., IBM Corp., Armonk, NY, USA) and a *P* < 0.05 was considered statistically significant.

## Results

A total of 15 (14 female and one male) patients with a median (range) age of 36 (26–56) years were included in this study. The main complaints were UUI and frequency in 13 and two patients, respectively. Table 1 lists baseline patients’ demographic data.

**Table 1.** Patients’ baseline demographic data.

Variable	Value
Age, years, median (range)	36 (26–56)
Sex, *n* (%)	
Male	1
Female	14 (93)
Symptoms, *n* (%)	
UUI	13 (86.8)
Frequency	2 (13.4)
Duration of symptoms, months, median (range)	24 (12–48)
History, *n* (%)	
Previous caesarean section	5 (33)
Q_max_, mL/s, median (range)	23(13–43)
PVR, mL, median (range)	6 (0–114)

For the primary endpoint, no patient developed haematuria, sepsis, severe suprapubic pain or retention over the study period. Two, two, and three patients developed UTI after 1, 2 and 3 months, respectively. Also, three and two patients developed asymptomatic microscopic haematuria after 1 and 2 months, respectively. The results of urine analysis and urine culture at the baseline and follow-up visits are listed in Table 2.

**Table 2.** Urine analysis and urine culture results during the study period.

Variable, *n*	Baseline	After 1 month	After 2 months	After 3 months
Urine analysis:				
Pyuria	0	2	2	3
AMH	0	3	2	0
Urine culture:				
Positive	0	2	2	3
Organism	0	*E.coli*	*E.coli*	*E.coli*

AMH: asymptomatic microscopic haematuria.

Compared with baseline, all domains of the OABSS including urgency, UUI, daytime frequency, nocturia, and total OABSS showed statistically significant improvements at 1 and 2 months of follow-up (*P* < 0.05). Nevertheless, at 3 months, only the nocturia score showed a statistically significant improvement (*P* = 0.02; Table 3). Seven (46.6%) and 12 (80%) patents were totally dry at 1 and 2 months, respectively. None of the patients had chronic retention requiring CIC. The median (range) Q_max_ increased during follow-up and reached statistical significance only after 2 months (*P* = 0.04). There was no statistically significant difference in PVR between the baseline and follow-up data (Table 4).

**Table 3.** OABSS at baseline and follow-up visits.

	Score, median (range)						
Score domains	Baseline	1 month	2 months	3 months	*P*1	*P*2	*P*3
Urgency	4.8 (4–5)	3 (0–5)	1.4 (0–5)	4.7 (0–5)	0.007	0.001	0.3
UUI	4.2 (0–5)	1 (0–5)	0.9 (0–5)	4.1 (0–5)	0.003	0.002	0.7
Daytime frequency	1.4 (0–2)	0.4 (0–2)	0.13 (0–1)	1 (0–2)	<0.001	0.002	0.8
Nocturia	2.7 (1–3)	1 (0–3)	1 (0–3)	2 (1–3)	0.001	0.001	0.02
Total score	14 (8–15)	5 (0–13)	1 (0–14)	12 (7–15)	0.001	0.001	0.1

*P*1: 1 month vs baseline; *P*2: 2 months vs baseline; *P*3: 3 months vs baseline. All comparison were done using Wilcoxon matched-pair signed-rank test.

**Table 4.** Q_max_ and PVR at baseline and follow-up visits.

Variable, median(range)	Baseline	1 month	2 months	3 months	*P*1	*P*2	*P*3
Q_max_, mL/s	23 (13–43)	24 (16–48)	30 (13–56)	26 (17–41)	0.07	0.04	0.2
PVR, mL	6 (0–114)	3 (0–80)	0 (0–50)	0 (0–67)	0.4	0.2	0.09

*P*1: 1 month vs baseline; *P*2: 2 months vs baseline; *P*3: 3 months vs baseline. All comparison were done using Wilcoxon matched-pair signed-rank test.

## Discussion

OAB can result from DO due to a neurological disorder (neurogenic type) in cases of spinal cord injuries and multiple sclerosis, but sometimes no definite cause of DO can be identified (idiopathic type) [1].

In cases of refractory OAB or intolerability to pharmacotherapy, intradetrusor injection of BoNT-A is recommended. It is licensed in Europe to treat OAB with persistent or refractory UUI in adults of both genders [3]. It is associated with significant improvement of the clinical manifestation with subsequent improvement of all aspects of patients’ QoL. After 3 months of BoNT-A injection, UUI episodes/day were halved and the number of micturitions/day reduced by more than two. A total of 22.9% of the patients were fully dry [10]. Nevertheless, it is done under anaesthesia with a special injection needle and associated with a substantial risk of infection, haematuria, pain, bladder injury, and an increase in PVR that may require CIC [11].

In a randomised controlled trial that compared BoNT-A injection 100 U to solifenacin, similar rates of improvement in UUI over the course of 6 months were reported. Patients who received BoNT-A were more likely to be cured of UUI (27% vs 13%, *P* = 0.003), but they had higher rates of urinary retention during the first 2 months (5% vs 0%) and of UTI (33% vs 13%). Patients taking antimuscarinic were more likely to have dry mouth [12].

On the other hand, intravesical instillation of BoNT-A in the bladder is not effective due to the high molecular weight of BoNT-A (150 kDa) making it difficult for it to pass through the urothelial barrier and reach the sub-mucosal nerve plexus to elicit an effect [4]. To overcome this barrier, intravesical instillation of BoNT-A formulated with liposome (lipo-botulinum toxin) to enhance its absorption was evaluated in a prospective, multicentre, double-blind, randomised trial. It was associated with decreases in OAB symptoms without side-effects [13].

Use of shockwaves was reported to increase cell membrane permeability to molecules up to 2 × 10^6^ molecular weight via generation of shear stress [8]. In a rat model, LESWs were shown to increase urothelial permeability and facilitate intravesical BoNT-A delivery into the detrusor muscle [4].

In the present study, we used LESWs to enhance delivery of BoNT-A into the detrusor muscle in a small number of patients with refractory OAB. Diagnosis was confirmed by urodynamic diagnosed DO and OABSS. BoNT-A 100 IU was used as a standard dose of injection. We chose this particular dose based on AUA guidelines for idiopathic DO [14]. The dose and the frequency of the LESWs were the same as that used in the treatment of erectile dysfunction and interstitial cystitis [15].

After 1 month of treatment, all domains of the OABSS were significantly reduced. Similarly after 2 months, the successful results of the LESWs-delivered BoNT-A were maintained. However, after 3 months, the scores of all OABSS domains were decreased but this was not statistically significant except for the nocturia domain. Also, 44.6% and 80% of patients became dry after 1 and 2 months, respectively. These results are comparable to previously reported data. Visco et al. [12] reported that 27% of patients were dry after 6 months of intradetrusor BoNT-A injection. In another single-blind, randomised, paralleled, actively controlled trial comparing the outcome of different sites of intradetrusor BoNT-A injection, 70–76% were dry after 12 months of follow-up [16]. In the present study, only 6.6% were dry after 3 months of treatment. This may raise concerns about the less durable outcome of LESWs-enhanced BoNT-A delivery in comparison to the intradetrusor injection with a shorter time to request re-treatment (2–3 vs 6 months) [10].

For the adverse effects, 13.3%, 13.3% and 20% developed UTI after 1, 2 and 3 months, respectively. Visco et al. [12] reported 33% of patients having UTIs following BoNT-A injection. In a systematic review published by Mangera et al. [3], the reported UTI complications with BoNT-A injection ranged between 13% and 44%. In the present study, there was no significant increase in PVR during the study period. After 1 month, three cases had a PVR of >50 mL, whereas after 2 and 3 months the PVR was >50 mL in one patient each. None of the patients had a PVR of >100 mL or required CIC. The reported range of increased PVR of >200 mL with intradetrusor BoNT-A injection is 27–43% [17,18]. The rate CIC following BoNT-A injection was reported to be 19–35% [19,20].

Although this is the first clinical trial to assess the impact of LESWs in improving delivery of BoNT-A into the bladder wall in humans with refractory OAB, it has many limitations. The main limitation was the small number of cases. But this is because of being a preliminary pilot study to assess the safety and efficacy of the treatment strategy. Only a dose of 100 IU of BoNT-A was studied and no escalation of the dose was tried. This may explain the less durable outcome of the treatment in comparison to intradetrusor injections. But our selection of this dose was based on previous results, where 100 IU was found to confer similar improvements in QoL as well as UI episodes as higher doses but with a lower rate of urinary retention (18% vs 25%) [3]. Moreover, the LESWs technique needs optimisation of the number of shockwaves delivered, rate of shockwave application, and number of sessions. Further studies including more cases with optimisation of the technique are warranted before comparing this new technique with other types of treatment for patients with refractory OAB.

## Conclusions

The new technique of intravesical instillation of BoNT-A and LESWs for the treatment of refractory OAB is safe and effective for 2 months. The effect is not durable due to the inadequacy of the dose of BoNT-A and/or non-optimised LESW technique. Much more study is required to improve the results of this new technique through optimisation of the dose of BoNT-A and the technique of LESW.
